# Sacubitril/Valsartan and Cognitive Outcomes in Heart Failure With Reduced Ejection: A New Pharmacological Indication?∗

**DOI:** 10.1016/j.jacadv.2023.100388

**Published:** 2023-06-30

**Authors:** Michele Correale, Massimo Iacoviello, Natale Daniele Brunetti

**Affiliations:** a Cardiothoracic Department, Policlinico Riuniti University Hospital, Foggia, Italy; b Department of Medical and Surgical Sciences, University of Foggia, Foggia, Italy

**Keywords:** angiotensin receptor-neprilysin inhibitor (ARNI), chronic heart failure, cognitive impairment, HFrEF, sacubitril

**Figure:**
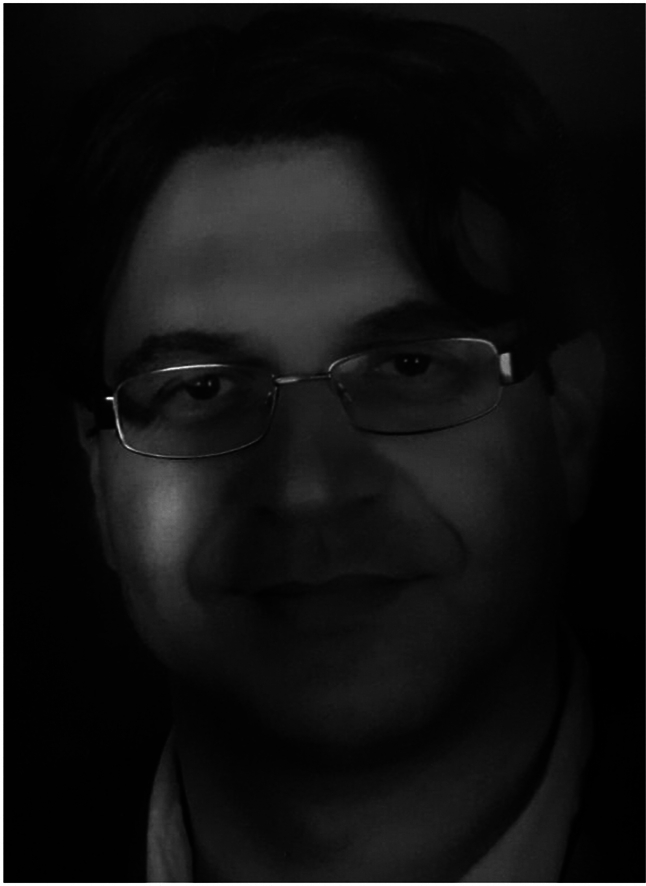

Comorbidities are a major issue in heart failure (HF), leading to frequent hospitalization and high mortality. Comorbidities may be cardiovascular and non-cardiovascular.^1^ Cognitive impairment (CI) is common in older adults with HF. The spectrum of CI in HF varies from delirium to isolated memory or non-memory-related deficits to dementia.^2^ CI is highly prevalent in both HF with reduced ejection fraction (HFrEF) (81%) and HF with preserved ejection fraction (77%) patients.^3^ Indeed, the exact prevalence and incidence of CI in these patients is uncertain. This is partially because of varying definitions and tools used to identify CI. Several factors may contribute to CI in HF, but hypertension, atrial fibrillation, stroke, and impaired hemodynamics are the most relevant. Cerebral hypoperfusion, disruption of blood-brain barrier, oxidative damage, platelet hyperreactivity, brain-endothelium inflammatory activation, and adrenergic system alterations are mechanisms.^3^ Assessment of cognitive functioning should be part of routine clinical examination in HF.

In HF, renin-angiotensin-aldosterone system blockade has been further developed by the introduction of the angiotensin receptor-neprilysin inhibitor sacubitril/valsartan, that combines renin-angiotensin-aldosterone system inhibition with the antagonism of neprilysin (NEP), boosting the positive effects of natriuretic peptides.^4^ Sacubitril/valsartan enriched the pharmacological approach to HFrEF, due to the positive effects on mortality,^5^ partly mediated by left ventricular reverse remodeling^6^^,^^7^ and by right ventricle function improvement.^8^^,^^9^ As NEP also impacts the degradation of amyloid-β and there is an ongoing concern about the effect of sacubitril/valsartan on cognitive function when used long term.

In this issue of *JACC: Advances*, Grewal et al^10^ present an interesting propensity-matched, retrospective cohort study using data from the TriNetX Analytics Research Network. The aim of the study was to evaluate the incidence of neurocognitive diagnoses, including cognitive decline, dementia, and Alzheimer’s disease. Three-year cognitive outcomes in patients with HFrEF who were switched to (or started) sacubitril/valsartan between 2015 and 2019, without further exposure to angiotensin-converting enzyme inhibitors (ACEIs) or angiotensin receptor blockers (ARBs), were compared to patients receiving ACEI and/or ARB exclusively during the same period. Data were obtained from TriNetX, an electronic health records network that provides access to anonymous electronic medical records from approximately 88 million patients receiving care at 58 health care organizations. For this study,^10^ data from 41 health care organizations in the United States were included. TriNetX provides demographics, clinical data (including laboratory results and medications), and standardized coding systems (International Classification of Diseases-10th Revision-Clinical Modification [ICD-10-CM] and Current Procedural Terminology codes for diagnoses and procedures, Logical Observation Identifiers Names and Codes for vital signs and laboratory values, and RxNorm for medications). Overall, the study included 11,313 adults with HFrEF (I50.2 or I50.4) who started or received sacubitril/valsartan between 2015 and 2019 (without any subsequent exposure to ACEI/ARB) and 11,313 matched patients who received ACEIs or ARBs exclusively during the same period. The article has interesting results. The use of sacubitril/valsartan in patients with HFrEF was associated with a lower incidence of neurocognitive disorder diagnoses (cognitive decline, dementia, and Alzheimer’s disease) at 3 years, compared to the use of ACEI/ARB. These findings were consistent in subcohorts of men and women, as well as in White and Black patients.

Grewal et al^10^ hypothesized that the incremental benefit of sacubitril/valsartan over ACEI or ARB on functional capacity and cardiac function^11^ may help prevent cognitive decline in patients with HFrEF, in conjunction with the neutral (and potentially beneficial) effect on amyloid β accumulation as shown in presented PERSPECTIVE (A Multicenter, Randomized, Double-blind, Active-controlled Study to Evaluate the Effects of LCZ696 Compared to Valsartan on Cognitive Function in Patients With Chronic Heart Failure and Preserved Ejection Fraction) trial.^12^ Furthermore, sacubitril/valsartan may improve cerebral perfusion and decrease pro-inflammatory cytokines contributing to the lower rates of neurocognitive disorders in patients with HFrEF. The data are in line with PARADIGM-HF (Prospective comparison of Angiotensin Receptor–Neprilysin Inhibition (ARNI) with ACE inhibitor to determine impact on Global Mortality and Morbidity in Heart failure trial)^5^ as 3-year all-cause mortality was lower in the sacubitril/valsartan compared to the ACEI/ARB cohort, and the annual rate of dementias was similar to both PARADIGM-HF arms.^13^

Although the authors report several limitations of this retrospective study (the incidence estimates of new cognitive disorders based on diagnostic codes and not on quantitative diagnostic tools; the entry criteria for the study included a diagnostic code-based definition for HFrEF and did not include quantitative left ventricle ejection fraction; in these cohorts with high mortality, the authors were not able to conduct a competing risks analysis because of limitations of the online analytics platform), the main limitation of this retrospective study is that the authors do not have serial cardiac or brain imaging, functional test, or biomarker data to support their hypotheses.

Results from this study suggest that sacubitril/valsartan is not associated with worse cognitive outcomes as initially hypothesized on the basis of theoretical concerns about NEP inhibition in the brain, but that, in fact, it may be beneficial for cognitive function among patients with HFrEF. However, in view of experimental studies in which sacubitril/valsartan increased the risk of Alzheimer's disease in control rats^14^ and caused a deleterious effect on cognitive function in a colchicine-induced Alzheimer's disease rat model,^15^ the efficacy of sacubitril/valsartan for the prevention of cognitive decline in patients with HFrEF and the identification of the pathophysiologic mechanisms need further prospective studies with additional information such as cardiac and brain imaging or biomarkers.

## Funding support and author disclosures

The authors have reported that they have no relationships relevant to the contents of this paper to disclose.
